# Hepatitis E and blood donation safety in selected European countries: a shift to screening?

**DOI:** 10.2807/1560-7917.ES.2017.22.16.30514

**Published:** 2017-04-20

**Authors:** Dragoslav Domanović, Richard Tedder, Johannes Blümel, Hans Zaaijer, Pierre Gallian, Christoph Niederhauser, Silvia Sauleda Oliveras, Joan O’Riordan, Fiona Boland, Lene Harritshøj, Maria São José Nascimento, Anna Rita Ciccaglione, Constatina Politis, Cornelia Adlhoch, Benoit Flan, Wahiba Oualikene-Gonin, Guy Rautmann, Paul Strengers, Patricia Hewitt

**Affiliations:** 1 European Centre for Disease Prevention and Control (ECDC), Stockholm, Sweden; 2 Hepatitis E Study Group, Joint PHE/NHSBT Blood Borne Virus Unit, PHE, Colindale, London, United Kingdom; 3 Paul-Ehrlich-Institute, Federal Institute for Vaccines and Biomedicines, Virus Safety Section, Langen, Germany; 4 Sanquin, Blood-borne Infections & AMC, Clinical Virology, Amsterdam, the Netherlands; 5 Etablissement Français du Sang, Saint-Denis, France; 6 Interregionale Blood Transfusion SRC, Berne, Switzerland; 7 Transfusion Safety Laboratory, Banc de Sang i Teixits, Barcelona, Catalonia, Spain; 8 Irish Blood Transfusion Service, Dublin, Ireland; 9 Rigshospitalet, Department of Clinical Immunology, Copenhagen, Denmark; 10 University of Porto, Faculty of Pharmacy, Porto, Portugal; 11 National Health Institute, Viral Hepatitis Division, Department of Infectious Diseases, Rome, Italy; 12 Hellenic Coordinating Haemovigilance Centre, Athens, Greece; 13 LFB Biomedicaments, Biological Safety Surveillance, Courtaboeuf Cedex, France; 14 Agence nationale de sécurité du médicament et des produits de santé, Saint-Denis Cedex, France; 15 European Directorate for the Quality of Medicines and HealthCare, Strasbourg, France; 16 International Plasma Fractionation Association, Amsterdam, Netherlands; 17 NHS Blood and Transplant, London, United Kingdom

**Keywords:** hepatitis E virus, blood donation, transmission, transfusion, screening

## Abstract

The public health implications of hepatitis E virus (HEV) in Europe have changed due to increasing numbers of hepatitis E cases and recent reports of chronic, persistent HEV infections associated with progression to cirrhosis in immunosuppressed patients. The main infectious risk for such immunosuppressed patients is exposure to undercooked infected pork products and blood transfusion. We summarised the epidemiology of HEV infections among blood donors and also outlined any strategies to prevent transfusion-transmitted HEV, in 11 European countries. In response to the threat posed by HEV and related public and political concerns, most of the observed countries determined seroprevalence of HEV in donors and presence of HEV RNA in blood donations. France, Germany, Spain and the United Kingdom (UK) reported cases of transfusion-transmitted HEV. Ireland and the UK have already implemented HEV RNA screening of blood donations; the Netherlands will start in 2017. Germany and France perform screening for HEV RNA in several blood establishments or plasma donations intended for use in high-risk patients respectively and, with Switzerland, are considering implementing selective or universal screening nationwide. In Greece, Portugal, Italy and Spain, the blood authorities are evaluating the situation. Denmark decided not to implement the HEV screening of blood donations.

## Background

Hepatitis E is a liver disease caused by infection with a small, non-enveloped, single-stranded RNA virus known as hepatitis E virus (HEV). Of four major HEV genotypes which infect humans, genotypes 1 and 2 are endemic and responsible for waterborne epidemics. Genotypes 3 and 4 are associated with zoonotic HEV infections transmitted to humans through consumption of raw or undercooked infectious pork and game products, and very rarely shellfish, or by contact with infected animals. The thermal resistance of HEV is relatively high in food products. The virus is successfully inactivated at food internal temperatures > 71 °C for at least 20 min [1]. Some people are unaware that gammon, sausages and salami may be cured but not cooked and therefore fall into the category of ‘uncooked meat’. Transmissions of HEV through transfusion and transplantation have also been reported [2,3].

A substantial increase of locally acquired HEV cases is observed across Europe where HEV genotype 3 infections, originating from animal reservoirs, are predominant and have become a common cause of acute viral hepatitis [4,5]. To analyse the trend in the incidence and prevalence of HEV infection in Europe, there is a need for harmonised case definition, surveillance system and testing algorithms [5]. HEV genotype 3 infection is commonly asymptomatic or mild and self-limiting without chronic sequelae [6]. Acute phase viraemia typically persists for 6 to 8 weeks, and because most cases are asymptomatic, it is possible for infected blood donors to donate while viraemic. A high frequency of viraemic donations, of up to 1:726 [7], has been detected in several European countries using nucleic acid testing (NAT). The number of notified transfusion-transmitted HEV (TT-HEV) infections has until now been very low, probably due to under-reporting and under-recognition mainly because of asymptomatic infections in transfusion recipients. All types of blood components including solvent–detergent (SD) treated plasma have been implicated in transmission. One United Kingdom (UK) study [8] retrospectively screened blood donations in pools of 24 samples and showed that one in 2,830 blood donations was HEV RNA positive. Follow-up of transfused HEV RNA positive blood components showed a 42.0% transmission rate, with transmission probability linked to viral load and absence of anti-HEV antibodies. Although HEV RNA was detected in plasma fractionation pools from Europe, Asia and North America, HEV transmission through plasma-derived medicinal products has not been observed [9]. The European Medicines Agency published a reflection paper on viral safety of plasma derived medicinal products with respect to HEV [10].

HEV genotype 3-related severe cases of hepatitis and chronic liver disease, occasionally leading to cirrhosis, have been reported in immunosuppressed transplant recipients, patients with haematological disorders and patients with underlying liver disease. Such patients are exposed to HEV primarily via daily dietary sources. Exposure to HEV through blood components is lower than dietary exposure in patients who have limited transfusion needs, but the risk of transmission through transfusion rises in the multi-transfused. One model estimates that receiving blood components from 13 donors carries a similar risk to 1 year of dietary exposure [11]. Prolonged viraemia and chronic liver injury can often be resolved by administration of ribavirin or reducing the level of immunosuppression [12]. The latter is challenging in haematological patients suffering from graft vs host disease and requiring more aggressive immunosuppressive therapy.

The European Pharmacopoeia requires HEV RNA screening of plasma pools for the production of SD plasma [13]. In response to a threat posed by HEV to transfusion safety and related public, political and reputational concerns, Ireland and the UK respectively have also implemented universal or selective (screening of donations for immunosuppressed recipients) screening of blood donations for the presence of HEV RNA, and others are considering doing so. Hepatitis E infection is not currently reportable under the provisions of European Union (EU) legislation [5].

Here we summarise the epidemiology of HEV infections among blood donors, along with that of reported cases of hepatitis E among patients, and also outline any strategies to prevent TT HEV, in 11 European countries (in alphabetical order) that were discussed during a European Centre for Disease Prevention and Control (ECDC) expert meeting in Lisbon, Portugal in May 2016.

## Hepatitis E virus and blood donations in selected European countries

### Denmark

Denmark has a large pig-farming industry and associated production of pig products. An investigation among pig herds in 2010 found HEV genotype 3 in the stools of more than 50% of herds investigated [14]. In 2013, a seroprevalence study among Danish blood donors showed 10% to 20% anti-HEV IgG positivity, depending on the assay used, and found an increase with age. Compared with seroprevalence studies of samples from Danish donors in 2003 and 1983, the authors found a declining prevalence corresponding to a birth cohort phenomenon [15].

In a Danish nationwide investigation, ca 25,000 donations from 2015 were screened for HEV RNA by Single Donation nucleic acid testing (NAT) (Grifols). The prevalence of HEV RNA positive donations was 1:2,331 (0.04%) [16], consistent with data from other northern European countries (Table). Positive donations had a median viral load of 13 IU/mL in a range from unquantifiable to 920 IU/mL [16]. Look-back studies of living recipients found no evidence of TT HEV infections [16]. Based on these data, the Danish Society of Clinical Immunology does not recommend the screening of blood donations for HEV in Denmark [17].

### France

HEV RNA NAT screening (pools of 96 samples) of plasma donations for subsequent solvent–detergent treatment in the period 2012/13 showed an HEV RNA positive detection rate of 0.04% (24/53,234) or 1:2,218 donations [18]. Most samples (22/24) from viraemic donors were negative for anti-HEV IgG and IgM. HEV genotype 3 was detected with viral loads from 468 to 5.1 10^6^ IU/mL. Recent data obtained from plasma donations screened by minipools (6 samples) indicate a higher rate of positive donations (ca 1:1,000). A seroprevalence study of 10,569 blood donations collected in mainland France and three overseas territories gave overall IgG (Wantai) prevalence 22.4% (range 8.0% to 86.4%) [19]. Significant geographical difference and hyperendemic areas in the southern part of France were found. IgG HEV seropositivity was associated with increasing age, eating pork meat, pork liver sausages, game meat, offal, and oysters, while drinking bottled water appeared protective. IgM seroprevalence was 1% (0–4.6%) [19]. Between 2006 and 2013, 16 cases of TT HEV genotype 3 were reported, mostly in immunocompromised solid organ transplant recipients as well as patients with haematologic malignancies under chemotherapy treatment, and confirmed by viral strain comparison. All types of blood components, including plasma treated by amotosalen and ultraviolet illumination, were implicated in transmission events [20]. Five cases of chronic HEV infection required ribavirin treatment [21]. Since 2013 the fraction of plasma collected by the French Blood Service that is intended for use in high-risk patients is screened for HEV RNA. NAT screening of blood donations is under consideration by the French health authorities.

### Germany

There has been a marked increase of notified cases, from 670 in 2014 to 1,267 in 2015 [22]. The reason for this increase is unknown, but could be due to growing awareness and testing by physicians. Studies in Germany found from 1:679 to 1:4,252 blood donations to be HEV RNA positive [23-25]. So far, five TT HEV infections have been reported in the German haemovigilance database, two in 2013 and three in 2014 [26]. Four cases were asymptomatic. In one case, HEV-infection was considered a co-factor leading to serious complications in a haematopoietic stem cell transplant patient suffering from severe graft vs host disease [27]. An analysis of transfusion-associated cases from 2015 has not been finalised, but three probable or confirmed TT cases were reported. One lymphoma patient receiving HEV RNA-positive transfusions after transplantation developed chronic hepatitis E. Two cases were asymptomatic. One additional case is still being investigated. Monitoring of immunocompromised patients, especially transplant recipients, for HEV infection is recommended by the German Advisory Committee Blood (Arbeitskreis Blut) [28]. Screening of blood donors has not been recommended but is under discussion [28]. As of December 2016, six blood establishments in Germany notified the German Competent Authority that they have commenced voluntary screening of blood donations for HEV RNA.

### Greece

In a recent study [29], archived blood samples from 1,835 blood donors, and recent samples from 249 thalassaemic patients from nine Greek regions were examined for anti-HEV antibodies using commercial ELISA and immunochromatographic tests. HEV RNA was tested for using the Procleix HEV (Grifols) assay in 1,813 blood samples from 1,670 blood donors and 143 thalassaemic patients. HEV IgG antibodies were present in 2.9% of blood donors. Seroprevalence was higher in older donors (5.9% in those aged > 50 years vs 1.8% in younger donors). The highest seroprevalence of 13.3% among male blood donors was found in Heraklion, Crete. Seroprevalence in thalassaemic patients was 3.6%. Results of HEV RNA testing among donors are not yet available. The data showed that the current seroprevalence of HEV in blood donors was lower in comparison to other European regions but showed an increasing trend. Donors’ age and sex were significant factors affecting the prevalence estimates. Most of the cases were imported from endemic countries outside Europe although the local acquisition of HEV requires further investigation in Heraklion.

### Ireland

Ireland implemented universal HEV individual-donation nucleic acid test (ID-NAT) screening of blood donations for an initial 3-year period from January 2016. By the end of April 2016, 47,229 donations had been screened using the Procleix HEV assay (Grifols). There were 27 initially reactive donations (0.057%), of which 16 (0.034%) were repeat reactive (RR) and 11 non-repeat reactive (NRR; 0.023%). Of the 16 RRs, 15 were confirmed using PCR testing (RealStar RT-PCR kit, Altona) and/or by serology (Wantai IgM and IgG assays). One RR sample was negative by PCR and serology but on follow-up 34 days later was PCR and IgM reactive. Overall, the majority of donors (11/15) were viraemic and seronegative at index donation. Results of genotyping are available so far for two confirmed cases which are genotype 3, phylogenetic group 2. Of the 11 NRRs, one donation was confirmed as an HEV case who subsequently seroconverted 70 days later with IgM and IgG reactivity. Eight NRRs have been confirmed as false positives, and two were awaiting follow-up testing at the time of writing this report. Of the 17 confirmed cases that occurred randomly across Ireland, 15 were male, and two were female. Episodic periods of reactivity with high levels of confirmed cases in January, early February, and mid-April were observed. The HEV RNA prevalence in Irish donations is currently 1:2,778, which is higher than expected from the previous study (anti-HEV IgG positive 5.3% in 2012 and an HEV RNA positivity of 1 in 5,000 donations in the period from December 2013 to June 2014 [30].

### Italy

The seroprevalence of hepatitis E in the Italian general population was analysed in six of 20 Italian regions and ranged from 1.5% to 2.9% [31-37]. Most of these studies were conducted before 1999. From 2007 to 2016, 144 cases of acute hepatitis E were notified to the Italian Surveillance System for Acute Viral Hepatitis [38]. Among 144 cases, 122 (84.7%) were male with a mean age of 40 years, 81 (56.6%) were Italian. The virological surveillance of 139 acute non-A, non-B, non-C hepatitis (cases negative for hepatitis A, B and C) from 2004 to 2016 showed that 48 (34.5%) cases were due to HEV. Genotyping of HEV RNA-positive samples revealed that 55% (22/40) of patients were infected with genotype 1 and 45% (18/40) with genotype 3. The prevalence of HEV antibodies in blood donors from Abruzzo and Lazio, two regions of central Italy, was 48.9% (153/313) and 9.0% (9/100) respectively in 2014 [39]. In Lazio, the seroprevalence of 9.0% in the donor population in 2014 is significantly higher than previously reported in the general population (2.6% in 1996 and 2.9% in 2007), which strongly suggests an increasing trend of HEV infection in the general population over almost two decades. The very high HEV IgG prevalence in blood donors from Abruzzo indicates that HEV infection is commonly acquired in this area. Seroprevalence increased with age and was associated with consumption of raw dried pork liver sausages. Among the IgG positive blood donors (n = 153) from Abruzzo, two (1.3%) were positive for IgM, and two (1.3%) were positive for HEV RNA; genotype 3 (subtype 3c) [39]. A national survey in 2016–2017 will evaluate the prevalence of HEV infection in 10,000 blood donations in Italy (270 Blood Transfusion Centres from 20 Italian regions). The results of the survey will be considered in developing HEV prevention strategy.

### The Netherlands

Since 2012, between 2,000 and 4,000 Dutch plasma donations have been screened each month by PCR on pools of 96 donations for the presence of HEV. Overall 79/101,793 or 1:1,289 donations were confirmed HEV RNA positive. HEV RNA sequencing shows HEV genotype 3 subtypes is present in Dutch hepatitis E patients and Dutch pigs [7]. This silent outbreak of HEV appears to be strikingly benign (i.e. lack of morbidity in neonates/infants up to the age of 12 months, children, and pregnant women). Cases of chronic hepatitis E have been reported in haematological and organ transplant patients. The dietary routes of HEV transmission in the Netherlands have not yet been thoroughly investigated. Recently, 43 of 55 liver sausages and 12 of 15 liver pâté samples were found to be positive for HEV RNA by PCR [40]. The Dutch Food Safety Authority has confirmed these findings, and haematological and organ transplant patients are advised to avoid these food items [40]. Although blood and blood components are probably a minor source of HEV infection in the Netherlands compared with dietary exposure, the presence of HEV-positive donations in the blood supply has given rise to the expression of concerns about the safety profile of Dutch blood banking, such as indicated by the request of one academic hospital for the supply of HEV RNA-screened blood.

HEV RNA screening of all Dutch blood donations is planned to start in July 2017. To provide HEV-screened blood for at-risk patients, 40% of Dutch donations must be screened. Considering the costs and the complicated IT and logistical consequences of partial donor screening, universal HEV RNA donor screening is being considered as more feasible.

### Portugal

HEV studies in Portugal are part of the HEPeCONTROL project (60DT2) under European Economic Area (EEA) grants funding [41]. Sera from a representative cohort of the Portuguese population (n = 1,656) distributed by geographic location (all 20 districts in Portugal), and 5-year age group (ranging from 0 to 99 years of age) were collected between July 2015 and February 2016, and tested for the presence of anti-HEV IgG by EIA (recomWell HEV IgG, Mikrogen) [42]. An overall HEV IgG seroprevalence in the Portuguese population of 16% was found with seropositivity significantly increasing with age (p < 0.05). Also, plasma samples from blood donors (n = 2,115) to a Blood Transfusion Service in Portugal collected between July 2015 and January 2016 were tested individually for both anti-HEV IgM (recomWell HEV IgM, version 2015, Mikrogen) and HEV RNA, using two commercial real-time RT-PCR kits (ampliCube HEV 2.0, Mikrogen and RealStar HEV 1.0, Altona).

Among the 2,115 plasma samples, 7 (0.3%) were found to be positive for anti-HEV IgM but no RNA HEV was detected in any of the blood donors’ samples. HEPeCONTROL project has given a sufficient picture of HEV infection in Portuguese general population and blood donors necessary for a decision on the implementation of a HEV national prevention strategy in the future.

### Spain

In 2013, the prevalence of anti-HEV IgG among 1,082 blood donors from Catalonia was found to be 19.9% (Wantai) and 10.7% (Mikrogen). Screening of 9,998 samples by HEV ID-NAT yielded three real-time PCR-confirmed and IgM and IgG anti-HEV-positive donations with viral loads of 250, 564, and 2,755 IU/mL. The donation with highest viral load was genotype 3f. HEV RNA positivity rate was 1:3,333 donations (0.03%).

The first symptomatic TT HEV case in Spain was reported in 2015. The immunocompetent patient developed clinical and laboratory signs of acute hepatitis more than 1 month after transfusion of eight red cell units during and after surgery. Investigation showed that one transfused RBC unit was positive for HEV RNA with 100% identity with the recipient HEV RNA sequences. The implicated donor had occupational exposure in a sausage factory. Platelets from the same infected donation were transfused to a Hodgkin lymphoma patient who died shortly after transfusion [43]. Selective HEV screening of blood donations is under consideration taking into account logistical challenges. Blood banks from central and northern Spain plan to study HEV incidence.

### Switzerland

A study of 550 donors from canton of Vaud in western Switzerland using three different HEV IgG EIAs (MP Diagnostics, Dia.Pro and Fortress) showed prevalences of 4.9%, 4.2% and 21.8% respectively [44]. An overall anti-HEV IgG seroprevalence of 8.9% (Mikrogen Diagnostic) in 1,484 donors was determined in the canton of Zürich in eastern Switzerland [45]. A third study analysed 3,609 blood donors from all over Switzerland [46]. The HEV IgG (Wantai) prevalence ranged from 12.8% (24/188) to 33.6% (116/345), depending on the geographical region. In the northern regions and within the Alps, seroprevalence seems to be quite similar (range: 12.8–24.8%) (24/188 to 69/278). In Ticino, a southern region of Switzerland, prevalence is higher with an average of 33.6% (116/345). In some smaller localities in Ticino, a prevalence bordering on 50% (13/27) was observed. Unfortunately, to date, no incidence data for the Swiss general population or the blood donor population have been collected.

Avoidance of potentially contaminated food is recommended to protect at-risk patients from dietary HEV infections. It is also proposed to screen blood donations for HEV RNA on pools of 96 samples and monitor at-risk patients. Clinicians will also be informed of these specific measures in order to draw attention to future HEV infections in at-risk patients.

### United Kingdom

Seroprevalence of anti-HEV IgG in 2015/2016 was found to be 11% (n = 13,042) in England and 6% (n = 1,700) in Scotland. There has been a steady increase in the incidence of acute cases since 2010 associated with the emergence of a dominant clade (G3c). An increased anti-HEV IgG positivity in younger donors was observed in 2015/16. Surveillance data indicate a sudden increase in reported possible TT HEV cases in 2012 followed by a sustained rise from 2015 onwards. Such growth probably resulted from a heightened clinical awareness, following the formation of an HEV working group of the Advisory Committee on Safety of Blood, Tissues and Organs (SaBTO). Reported cases of possible TT HEV were acute clinical hepatitis in immunocompetent individuals and silent infection in immunosuppressed haematopoietic stem cell and solid-organ transplant patients. A proportion of investigated cases were shown not to be transfusion-associated. SaBTO recommended the provision of HEV-screened blood components for recipients of allogeneic stem cell transplants and solid organ transplants [47]; the blood services additionally provide HEV-screened components for neonates/infants up to the age of 12 months. A minimum of 30% of the blood supply is tested to meet current demand. As of May 2016, with blood donation screening using NAT RNA HEV test (pools of 24 samples), the number of confirmed RNA-positive samples was 83 of 113,306 (1:1,365) tested in England, 10 of 12,504 (1:1,250) tested in Scotland, and 2 of 4560 (1:2,280) in Wales. Look-back investigations are performed when blood donors are reported with acute HEV infection, but the donor HEV infection must be substantiated. The immediate previous donation of HEV RNA positive platelet donors is also subject to look-back. As of November 2016, the SaBTO has completed a review of HEV screening and its cost-effectiveness and has recommended extending the use of screened components to other recipient groups and a change to universal screening of blood donations from April 2017 [48]. The principal drivers for this decision were technical complexity and costs of a double inventory that was needed in selective screening.

## Discussion

The public health implications of HEV in Europe have changed recently due to the increasing numbers of hepatitis E cases and reports of chronic, persistent HEV infections associated with progression to cirrhosis in immunosuppressed patients. The main infectious risks for such immunosuppressed patients are dietary exposure to pork products and transfusion. In the last decade, an increasing number of HEV genotype 3-positive donations have been documented in several European countries (Table). Such growth probably resulted from a heightened clinical awareness, and following on from national blood safety discussions and measures in some countries.

**Table:** Prevalence of hepatitis E virus RNA positive donations, population of transplanted patients at risk, reported cases of transfusion-transmitted hepatitis E virus and screening of blood donations in 11 European countries

Country	HEV RNA positive donations	Population at risk		Reported TT HEV infections	Screening of blood donations			
		allo-HSCT [51] AN (AR/p10mp)	SOT [52] AN (AR/pmp)		Implemented	Under Consideration	In evaluation	Not recommended
**Denmark**	1:2,331(2016) [16]	144 (201 – 300)	356 (63.6)					x
**France**	1:2,218 (2012–3) [18]	1,724 (201 – 300)	5,141(79.6)	x		x^a^		
**Germany**	1:1,241 (2012) [24]	2,892 (> 300)	3,710 (44.9)	x		x^b^		
**Greece**	NA	169 (151 – 200)	171 (15.4)				x	
**Ireland**	1:2,778 (2016)	77(151 – 200)	246 (52.3)		x^c^			
**Italy**	NA	1,625 (201 – 300)	3,252 (53.2)				x	
**The Netherlands**	1: 726 (2016) [7]	1175 (> 300)	1,315 (78.3)			x ^d/e^		
**Portugal**	NA	137 (101 – 150)	739 (69.7)				x	
**Spain**	1:3,333 (2014) [53]	1,072 (201 – 300)	4,247 (90.2)	x			x	
**Switzerland**	NA	191 (201 – 300)	504 (61.5)			x		
**United Kingdom**	1:1,340–5,000 (2016)	1,602 (201 – 300)	4,561 (71.8)	x	x^e/f^			

allo-HSCT: allogeneic haematopoietic stem cell transplant patients; AN: annual number; AR: annual rate; HEV: hepatitis E virus; NA: not available, pmp: per million population; SOT: solid organ transplant patients; TT: transfusion-transmitted.

^a^ Testing part of plasma production for use in patient at risk.

^b^ Screening of all blood donations in some blood centres.

^c^ Universal screening.

^d^ Screening of plasma for the production of solvent-detergent treated plasma for clinical use.

^e^ Universal screening planned for 2017.

^f^ Selective screening.

Taking into account the prevalence of HEV infection in pigs and pork meat consumption in the EU [49,50] on the one hand, and the incidence of HEV viraemic blood donations (Table) on the other, it seems that for the general population, the risk of HEV transmission via food products is considerably higher than through blood transfusion. Therefore, from a public health perspective, eliminating the dietary risk of HEV transmission is the most effective intervention and would also improve blood safety. Until and unless the dietary risk is eliminated, TT HEV infection in immunosuppressed patients represents a preventable cause of serious morbidity and mortality and as such indicates a need to improve transfusion safety.

Approaches to increase blood safety for HEV are currently limited. Potential measures include: pathogen inactivation of blood components, which may not yet be sufficiently effective for certain non-enveloped viruses such as HEV; immunisation of patients at risk, which is not yet available and is unproven against intravenous challenge; and screening of blood donations, which is an available intervention but not widely implemented because of effectiveness constraints and costs. In the absence of the implementation of such measures, individuals at risk of developing severe consequences remain exposed to the risk of TT HEV infection.

Universal screening of blood donations has been implemented in Ireland and selective screening implemented in the UK in 2016. The majority of the other European countries assessed here, eight of 11, were at the time of the meeting investigating or considering the need to screen blood donations for the presence of HEV RNA. The UK and the Netherlands have, since the meeting, decided to implement universal screening in 2017, as the most cost-effective way of ensuring provision of HEV screened blood components for the recommended patient groups. Denmark has decided not to screen (Table).

The rationale for donation screening for any pathogen is particularly strong when there are specific recipient groups at risk of transfusion-transmitted infection. The population currently recognised to be at risk of TT HEV (recipients of solid organ and haematopoietic stem cell transplants, and other immunosuppressed patients) is relatively small in number (Table) but may receive substantial number of transfusions, which increases the probability of exposure and thus the risk of infection.

Justification of blood donation screening for HEV RNA becomes contentious if the risk from exposure of transfusion recipients to dietary sources is not eliminated. Equally, some TT infections which cause disease in the immunosuppressed, such as parvovirus B19, remain tolerated and unscreened. In transfusion practice, however, laboratory screening of some pathogens (i.e. cytomegalovirus) has been implemented, despite a sustained community exposure of transfusion recipients. Thus, it seems that the risk of non-transfusion exposure to HEV might not be critical for a decision to implement HEV RNA screening of blood donations.

If implemented, screening may be selective, performed on blood donations intended for transfusion to patients at risk, or universally applied to all blood donations and subject to continuing review. Selective screening might be technically demanding especially with the management of the blood component inventory and is not necessarily less costly.

The decision to screen blood donations should be based on an assessment of the risk of TT HEV in the susceptible population, as determined by the background prevalence of transmissible infection in the donor population and the susceptibility of recipients. Further, to assess the absolute benefit of screening, detailed knowledge about the incidence of hepatitis E in patients receiving HEV-negative blood components and those who receive unscreened blood components is required. The costs as well as the benefits also need to be taken into account. Defining the appropriate sensitivity level of NAT testing (pool size) is one of the key issues with an impact on the cost-effectiveness of routine screening.

Despite the uncertainties in the epidemiology of HEV, screening of blood donations for the presence of HEV RNA is clearly under consideration in several countries and has been currently implemented in two of those reviewed here. However, given what is known about the risks of dietary transmission of HEV infection, if implemented, the screening of blood donations should go hand in hand with raising clinicians’ awareness and strict dietary recommendation for patients at risk. Validation of NAT findings by seroconversion, sequencing of viral RNA genome using common sequence database, and follow-up of HEV cases among blood donors and patients may help to define the relative contributions of different routes of HEV infection in Europe. Ultimately, addressing the root cause for viraemic pigs entering the human food chain will be required to achieve control of this zoonosis.
